# Retrospective Analysis of Duodenal Injuries: A Comprehensive Overview

**DOI:** 10.4103/1319-3767.77247

**Published:** 2011

**Authors:** Sanjay Pandey, Ashutosh Niranjan, Shashank Mishra, Tarun Agrawal, Basant M. Singhal, Akhil Prakash, Prakash C. Attri

**Affiliations:** Department of Surgery, Subharti Medical College, Meerut, India

**Keywords:** Blunt trauma abdomen, duodenal injury, pancreatico-duodenal injury

## Abstract

**Background/Aim:**

Duodenal injury is an uncommon finding, accounting for about about 3 – 5% of abdominal trauma, mainly resulting from both penetrating and blunt trauma, and is associated with significant mortality (6 - 25%) and morbidity (30 - 60%).

**Patients and Methods:**

Retrospective analysis was performed in terms of presentation, management, morbidity and mortality on 14 patients of duodenal injuries out of a total of 172 patients of abdominal trauma attending Subharti Medical College.

**Results:**

Epigastric pain (100%) along with vomiting (100%) is the usual presentation of duodenal injuries in blunt abdominal trauma, especially to the upper abdomen. Computed tomography (CT) was diagnostic in all cases. Isolated duodenal injury is a rare finding and the second part is mostly affected.

**Conclusion:**

Duodenal injury should always be suspected in blunt upper abdominal trauma, especially in those presenting with epigastric pain and vomiting. Investigation by CT and early surgical intervention in these patients are valuable tools to reduce the morbidity and mortality.

Injuries to the duodenum account for approximately 3%-5% of abdominal trauma.[1–3] Blunt abdominal trauma as a result of direct blow to the epigastrium, mainly due to road traffic accident and sports trauma (bicycle handle injury), accounts for 25% of all duodenal injuries.[4,5] The remaining 75% are due to penetrating trauma.[1–3] Isolated duodenal injuries are very rare due to deep and relatively well-protected anatomical site of the duodenum. They are commonly associated with injuries of other abdominal or thoracic organs, including major vessels.[1–5] Contrary to other intestinal injuries which present with peritonitis and shock, the diagnosis of duodenal injuries is often delayed, contributing to high morbidity and mortality.

## PATIENTS AND METHODS

This study had been conducted at Subharti Medical College, Meerut, India, after obtaining the permission from the institutional ethics committee. This is a retrospective analysis of patients presenting with blunt abdominal trauma during January 2004 to December 2008. This study was conducted with the aim to establish the pattern of presentation of duodenal injuries, especially in blunt abdominal trauma, where the diagnosis is always a dilemma, so that a protocol of its management could be derived to minimize the morbidity and mortality. We analyzed 14 patients of duodenal injury out of 172 abdominal trauma patients admitted to our institution during the above period.

## RESULTS

**Figure 1:**
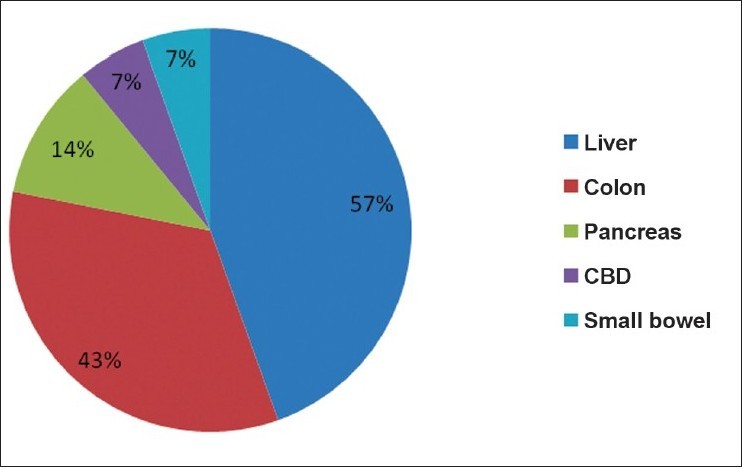
Pi-chart showing associated injury

**Figure 2:**
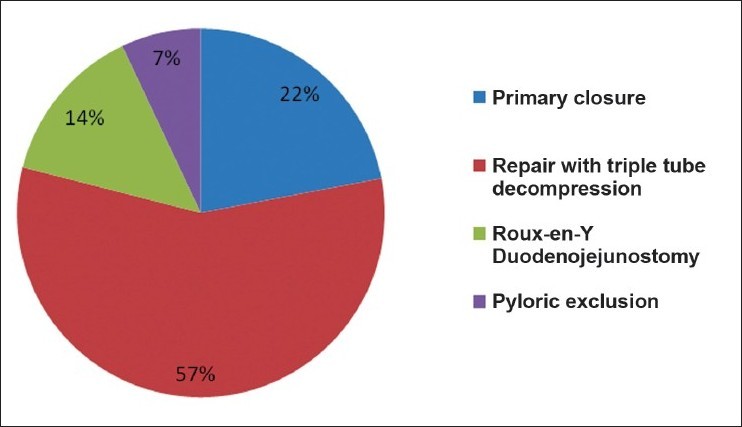
Pi-chart showing different surgical procedures

Duodenal injury was more prevalent due to penetrating injuries (57%) than blunt injuries (43%) of abdomen [Table 1]. Most of the patients of penetrating injury had reported to hospital within 6 hrs (*n*=6) while in blunt trauma they reported to hospital in between 12 and 24 hrs (*n*=4). We observed that epigastric pain (100%) with vomiting (100%) was the key presentation in this series, whereas back pain (36%), distension of abdomen (36%) and even peritonitis (43%) were encountered in less than half of the cases. The features of shock were seen in 57% of the cases [Table 1]. Peritonitis and distension of abdomen was present in those patients, who were admitted to the hospital after 24 hrs. Except for the history of blow to upper abdomen or fire arm injury followed by epigastric pain and vomiting, the clinical features were not consistent. Free air under the right dome of diaphragm was present in only 7% of cases, whereas CT findings of retroperitoneal edema and collection around duodenum and pancreas (43%) and retroperitoneal air (14%) was more prevalent [Table 2].Level of serum amylase above 300 IU was seen in 42% of cases. All patients were operated between 24 - 48 hrs after admission. Yellowish or chocolate discolorations of peritoneal tissues were encountered in almost all patients, who had been operated and duodenal injuries were present. The other operative findings were retroperitoneal hematoma near duodenum or pancreatic head extending to base of mesocolon in 86%, fat necrosis of retroperitoneal tissue or mesocolon in 50% and crepitation with bile stained fluid along the lateral margin of duodenum in 36% of cases. The second part of duodenum was affected more (58%) as compared to the other parts of duodenum and none of these patients had isolated duodenal injury. Liver (57%) was mostly affected as associated injury followed by colon (43%), pancreas (14%), CBD and small bowel (7% each) [Figure 1]. Majority of the patients (57%) were managed by closure of injury along with triple decompression, followed by primary closure (22%), Roux-en-Y duodenojejunostomy(14%) and pyloric exclusion(7%) [Figure 2].The post operative complications like duodenal leak, subphrenic abscess, wound dehiscence and chest infection were more in the patients who had duodenal injuries due to blunt trauma of abdomen. The average hospital stay was 10-14 days and 2 patients died post operatively. These patients had presented in the hospital after 24 hrs and had associated other organ injuries and had developed post operatively severe chest infections along with duodenal leak.

**Table 1:** Presenting symptoms and signs in duodenal trauma

Clinical features	Penetrating injuries (n=8)	Blunt injuries (n-6)	Total (n=14)
Pain in epigastrium	8 (57)	6 (43)	100
Pain in back	1 (7)	4 (29)	36
Vomiting	8 (57)	6 (43)	100
Shock	6 (43)	2 (14)	57
Peritonitis	4 (29)	2 (14)	43
Distension of abdomen	3 (22)	2 (14)	36

Figures in parenthesis are in percentage. Shock was defined on the presence of the following 3 criteria: pulse >90/min, systolic pressure <90 mmHg, urine output <25 ml/hr

**Table 2:** Different findings of investigations in duodenal trauma

Imaging study	Finding	No. of patients	%
X-ray (n=14)	Metallic foreign body	6	43
	Free air	1	7
USG (n=14)	Intra-peritoneal fluid collection	7 (Assault injuries)	50
CT scan Abdomen (n=14)	Extravasation of contrast	3	21
	Retroperitoneal air	2	14
	Retroperitoneal oedema	6	43
	Collection around duodenum/pancreas	6	43
	Intra-peritoneal collection	8	57
			

## DISCUSSION

Prompt diagnosis and efficient treatment of duodenal injury is crucial, with evidence demonstrated by Lucas and Ledgerwood in 1975 suggesting that a delay in diagnosis and treatment of more than 24 hrs after injury can increase mortality from 11% to 40%.[6] However, the diagnosis is difficult unless a high index of suspicion is maintained in all cases of abdominal trauma, which otherwise may lead to misdiagnosis or delay in diagnosis. Ultrasound can be performed initially to rule out other injuries to intra-abdominal organs and vessels but, is an inadequate test for pancreatico-duodenal area.[4] Currently, contrast enhanced CT (CECT) is the diagnostic test of choice in stable patients with blunt abdominal trauma. The presence of retroperitoneal extra luminal air on CT is an important sign of duodenal injury requiring surgical repair. In fact, in this way, it may be possible to demonstrate the extravasations of contrast media in the presence of laceration. However, in some cases even CT scan can be negative at admission, or subtle CT findings such as small amount of unexplained fluid and unusual bowel morphology can be underestimated and dismissed.[7–11] For these reasons, subtle findings on abdominal CT should be an indication for urgent laparotomy or explorative laparoscopy. In our study, all the patients were evaluated with plain X-ray of abdomen, ultrasonography and CT scan. We observed that the findings of CT were always significant as compared to other radiological investigations. Serum amylase level might be helpful, since persistently increased or rising level can be an indication of a lesion in the duodeno-pancreatic area. The treatment of duodenal injuries is based on the underlying etiology, severity of the injury, associated injuries to intra and extra-abdominal organ systems, and duration of delay in diagnosis.[12] Complications, such as fistula formation and post operative chest infection, are more common after the repair of duodenal injuries (2%-14%).[12] In this study, post operative complications, which were more in the duodenal injuries associated with blunt trauma, may be due to diagnostic dilemma leading to delay in intervention.

## CONCLUSION

Duodenal injuries should be suspected in blunt abdominal trauma patients presenting with epigastric pain and vomiting, and urgent CECT scan is strongly recommended. Immediate surgical intervention is an important factor to minimize post operative complications, so even subtle findings in CT scan are an important indication for surgery.
